# Analysis and Prediction of the Critical Regions of Antimicrobial Peptides Based on Conditional Random Fields

**DOI:** 10.1371/journal.pone.0119490

**Published:** 2015-03-24

**Authors:** Kuan Y. Chang, Tung-pei Lin, Ling-Yi Shih, Chien-Kuo Wang

**Affiliations:** 1 Department of Computer Science and Engineering, National Taiwan Ocean University, Keelung, Taiwan; 2 Department of Biotechnology, Asia University, Taichung, Taiwan; Nanyang Technological University, SINGAPORE

## Abstract

Antimicrobial peptides (AMPs) are potent drug candidates against microbes such as bacteria, fungi, parasites, and viruses. The size of AMPs ranges from less than ten to hundreds of amino acids. Often only a few amino acids or the critical regions of antimicrobial proteins matter the functionality. Accurately predicting the AMP critical regions could benefit the experimental designs. However, no extensive analyses have been done specifically on the AMP critical regions and computational modeling on them is either non-existent or settled to other problems. With a focus on the AMP critical regions, we thus develop a computational model AMPcore by introducing a state-of-the-art machine learning method, conditional random fields. We generate a comprehensive dataset of 798 AMPs cores and a low similarity dataset of 510 representative AMP cores. AMPcore could reach a maximal accuracy of 90% and 0.79 Matthew’s correlation coefficient on the comprehensive dataset and a maximal accuracy of 83% and 0.66 MCC on the low similarity dataset. Our analyses of AMP cores follow what we know about AMPs: High in glycine and lysine, but low in aspartic acid, glutamic acid, and methionine; the abundance of α-helical structures; the dominance of positive net charges; the peculiarity of amphipathicity. Two amphipathic sequence motifs within the AMP cores, an amphipathic α-helix and an amphipathic π-helix, are revealed. In addition, a short sequence motif at the N-terminal boundary of AMP cores is reported for the first time: arginine at the P(-1) coupling with glycine at the P1 of AMP cores occurs the most, which might link to microbial cell adhesion.

## Introduction

Antimicrobial peptides (AMPs) are potent drug candidates against microbial organisms such as bacteria, fungi, parasites, and viruses. AMPs, which play an important role in innate immune responses to microbes, are derived from a broad spectrum of species such as archaea, bacteria, plants, and animals [1, 2]. Lysozyme is the first AMP discovered in human tissues and body fluids about 90 years ago [3]; α-purothionins found in wheat is the first AMP of plants [4]; gramicidine, a mixture of several AMPs, is one of the earliest AMPs derived from bacteria, *Bacillus brevis* [5]. However, the discovery of penicillin, the super antibiotic, might have hindered the development of AMP researches in the mid-20th century. Not until 1980's, the AMP researches revived. Then more AMPs were found including cecropins of silk moths [6], sarcotoxins of flies [7], defensins of rabbit macrophages [8], and magainins of frogs [9]. Up to date, thousands of AMPs are available and more than a dozen AMPs have entered clinical trials [10].

AMPs with various sizes have been documented in the literature. They range from ten to hundreds of amino acids. It is common to see that some AMPs would nest on other longer sequences. Such findings attribute antimicrobial effects to certain key active segments or critical regions of AMPs. Here we list three well-studied cases as examples: (I). Human LL-37 of human cathelicidin antimicrobial peptide (CAMP) expressed in different cells such as neutrophils, mast cells, epithelial cells, and macrophages [11–14]. Human LL-37 which consists of 37 amino acids beginning with double leucine is known to be antimicrobial [15]. In addition, KR-12 with only 12 amino acids is the shortest active segment of human LL-37 (18–29) [16]. (II). Human lactotransferrin, also known as human lactoferrin (hLF), found in various body fluids such as milk, tears, saliva, and nasal mucus as well as neutrophils [17–19]. Both hLF and its short helical segment with 11 amino acids hLF(21–31) demonstrated potent antimicrobial activities [20]. Similar cases were also seen in non-human LFs and their short segments [21, 22]. (III). Histidine-rich human histatin secreted from parotid and submandibular salivary glands [23]. Human histatin 8 with 12 amino acids is the shortest antimicrobial segment derived from histatin, which is common to most of the histatin family including histatin 3, 4, 5, 6, 7, 9, and 10 [24].

How AMPs defeat microbes have attracted researchers’ attention. It is believed that AMPs, abundant in cationic residues like lysine and arginine and scarce in anionic residues like aspartic acid and glutamic acid, interact with the anionic membranes of microbes to form transmembrane pores, thus causing abruption of microbes [10]. Besides, AMPs may enhance the progress of phagocytosis or the recruitment of leukocytes [25], and could even alter the gene expression of microbes [26].

Several computational methods have been applied to predict AMPs. They include quantitative matrix, discriminant analysis, artificial neural network, neuro-fuzzy interference, hidden Markov model, support vector machine, random forest, quantitative structure-activity relationship, and feature selection method [2, 27–32]. Generally speaking, these models utilized various features such as amino acid composition, protein secondary structure, net charge, and peptide aggregation to predict whether protein sequences are AMPs.

However, little is done in computationally identifying the critical regions of AMPs. To explain the differences between predicting AMPs and predicting AMP critical regions, the analogy of gene prediction is used. The current AMP prediction is like predicting whether genomic DNA sequences contain genes, but not where genes are located. Identifying the critical regions of AMPs is like finding which regions of DNA sequences encode genes. Modeling AMP critical regions requires understanding not only the differences between AMPs and non-AMPs but also how critical and non-critical AMPs regions are transited. Here we use the AMP critical regions defined as the shortest segments of the nested AMP families retaining antimicrobial effects deduced from current experimental evidence to model AMP cores.

Among these computational approaches, the AMPA server developed an unconventional method using high-throughput substitution data of 12-mer bactenecin against *Pseudomonas aeruginosa* to identify the active AMP stretches [33]. This method assigned each amino acid a bactericidal propensity value, which is the average of the bacterial half-maximal inhibitory concentration (IC50) of all the 12 variants of bactenecin [34]. According to the bactericidal propensity values, arginine and lysine, the basic residues, were the most lethal amino acids against microbes; aspartic acid and glutamic acid, the acid residues, were the least lethal.

To our knowledge, this is the first study to systematically extract the critical regions of AMPs or the cores of AMPs, examine their properties, and employ conditional random fields (CRFs) with multiple features to model them. Several important features of the AMP cores are investigated, including amino acid composition, protein secondary structures, net charges, amphipathicity, conserved protein domains, gapless alignments to highly similar protein sequences, AMPA bactericidal propensity, and peptide aggregation.

## Materials and Methods

**Fig 1:**
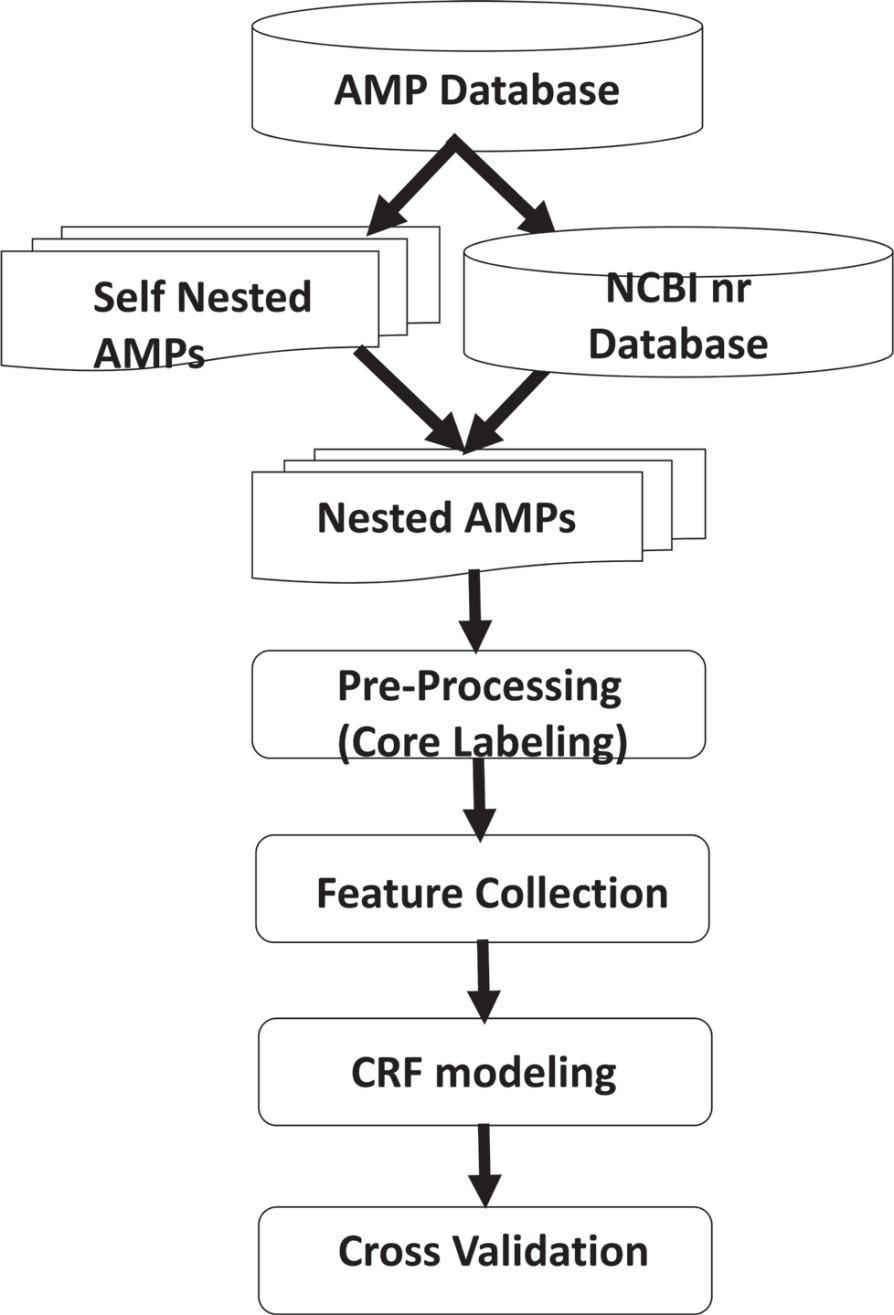
Flowchart of AMPcore. 2,497 non-redundant experimentally validated AMPs were obtained from CAMP release 2. 798 AMP nested families are recognized through self-comparison and their source proteins from NCBI nr database. The critical regions or cores of the nested families are then determined along with their features such as protein secondary structures and conserved domains. AMPcore are built using CRFs based on the nested AMPs and the performances are examined using 10-fold cross-validation.

The framework of AMPcore is shown in Fig. 1. Nested AMP families were first generated. Next, the critical and non-critical regions of AMPs along with their features were determined. Our models based on these features were then trained using CRFs and were evaluated by 10-fold cross-validation.

### Data collection of AMP critical regions

AMP critical regions are defined here as the shortest experimentally-validated segments of AMP proteins retaining antimicrobial function. In computational terms, AMP critical regions are the minimal substrings of AMP protein sequences supported by experimental evidence. It should be noted that shorter AMP cores which have not yet been found by experiments might exist.

The AMP critical regions in this study were determined by either nested AMP families or source proteins. 2,497 experimentally validated non-redundant AMPs were obtained from CAMP release 2 [2]. 158 nested AMP families were directly extracted from the experimentally validated dataset. In addition, each AMP sequence in the dataset was run against the NCBI non-redundant (nr) protein database using BLAST to search for its full-length source protein. The longer source proteins were also included in the nested families. Each nested AMP family thus contains at least one short AMP sequence and at least one AMP source protein. Those families with single members without valid source proteins, were not considered. 798 nested AMP families were collected in our final collection. Based on this collection, 510 representative nested AMP families were also generated by filtering out highly similar families with over 70% sequence identity using CD-HIT [35]. Such cutoff threshold was chosen by following Wang *et al*. [27].

The AMP critical regions of a nested AMP family can be described as follows: Let *P*_*m*_ = *P*_*m*,*1*_ *P*_*m*,*2*_ … *P*_*m*,*n*_ be a protein sequence with n residues. Given a nested AMP family *P* = {*P*_*1*_, …, *P*_*k*_} with k ≧ 2 and | *P*_*i*_ | = *n*_*i*_, the AMP critical region is determined by the shortest AMP *P*_*s*_.

$$P_{s}:=P_{s}\in P\wedge P_{s}\subseteq \overset{k}{\underset{i=1}{\cap }}P_{i}\wedge \left|P_{s}\right|=\underset{1\leq i\leq k}{\min}(\left|P_{i}\right|)$$

where *Ps* is a member of the family and also a substring of all the other AMP sequences in the family. In our study, 40 ≧ | *Ps* | ≧ 7. Outside *Ps* is considered to be non-critical for antimicrobial function.

**Fig 2:**
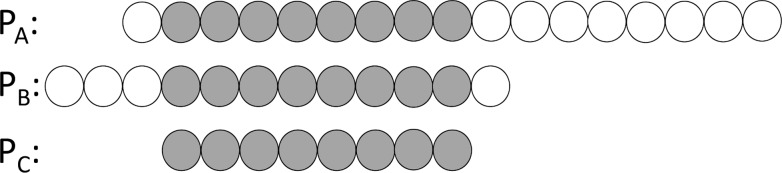
A simple diagram of a nested AMP family. This nested family has three AMPs: P_A_, P_B_, and P_C_. Each circle represents an amino acid. The circles in gray are the critical regions of the family, which are the overlapping residues shared by all the family members; those in white are the non-critical regions. Note: here a family has at least two members and one member with at least seven amino acids long would map to the critical region entirely.

The non-critical regions of a nested AMP family are determined once *Ps* is found. The longest sequence in the family marked with the critical and non-critical regions is utilized to train our model. Fig. 2 illustrates the concept of a nested AMP family.

### Conditional random fields

CRFs are a discriminative undirected probabilistic graphical model proposed by Lafferty *et al*. [36]. Both generative and discriminative models are probabilistic models. However, generative models such as hidden Markov models (HMMs) are based on the joint probability distribution P(*Y*, *X*) and discriminative models such as maximum entropy Markov models (MEMMs) and CRFs are based on the conditional probability distribution P(*Y* | *X*), where variables *X* and *Y* represent observations and labels respectively. The fundamental differences require generative models to model P(*X*), which is difficult when P(*X*) involves multiple attributes. Generally speaking, CRFs outperform traditional generative graphical models like HMMs because they can relax the strong independence assumptions made in generative models [36].

In addition, CRFs overcome the label bias problem which other discriminative models based on next-state classifiers like MEMMs suffer. Given a current state, these models determine which state to go next by comparing all outgoing transitions of the current state, not all transitions of the models. The per-state computation is biased in favor of the states with fewer outgoing transitions. Instead CRFs are designed to consider the entire label sequence given the observation sequence in terms of conditional probability.

We would like to address our problem as a sequence labeling problem. A typical sequence labeling problem is to predict a sequence of categorical labels *Y* = (y_1_, y_2_,…, y_n_) given a sequence of observations *X* = (x_1_, x_2_,…, x_i_). CRFs are known to excel in sequence labeling tasks such as part-of-speech tagging and text segmentation in natural language sequences [36, 37], image segmentation in video sequences [38], and gene finding [39], RNA secondary structural alignment [40], protein structural instability [41] and protein domain boundary in biological sequences [42].

To determine the most probable sequence of labels $\hat{Y}$, that is,

$$\overset{⌢}{Y}=\arg\underset{Y}{\max}P(Y|X)$$

CRFs use an exponential function to compute the conditional probability of labels with respect to observations as follows:

$$P(Y|X)=\frac{e^{{}^{\sum_{i=1}^{n}\sum_{j}\lambda_{j}f_{j}(y_{{}_{i-1}},y_{{}_{i}},x,i)}}}{Z(X)}$$

where index *i* represents position *i* in the sequence, *j* represents the *j*th observed feature, *λj* is a *j*th weight vector, and *f*_*j*_ is a *j*th feature vector which considers both a transition feature *tj*(*y*_*i-1*_, *y*_*i*_, *x*, *i*) between position *i*-1 and position *i* and a state feature *sj*(*y*_*i*_, *x*, *i*) at position *i* in the label sequence given the observation *x*. Z(X) is an observation-dependent global normalization function which enumerates all possible labels given the observation *x* as follows:

$$Z(X)=\sum_{j}^{}e^{\sum_{i=1}^{n}\sum_{j}\lambda_{j}f_{j}(y_{i-1},y_{i},x,i)}$$

To maximize the likelihood of $\hat{Y}$, the optimal λ is found using L-BFGS algorithm [43], a limited-memory quasi-Newton method, in the iterative training stage. CRFs guarantee to reach a global maximum likelihood, for they are globally conditioned on the observation *x*. More detail about the parameters in CRFs can be found in Lafferty *et al*. [36]. In our case, the conditional probability distribution treats protein sequences as the primary observations and critical/non-critical regions as the labels. Each residue of the protein sequence would be mapped to an associated label.

In this study, CRF++ version 0.58 was utilized [44]. The rules for training our CRF models were obtained from the template files.

### Observed Features

We examined eight different aspects of the critical regions of AMPs:

1. Protein primary structures
2. Protein secondary structures
3. Conserved protein domains
4. Short pairwise alignments
5. AMPA
6. Aggregation
7. Net charges
8. Amphipathicity

A sliding window was applied to study the features/observations of the critical and non-critical regions of AMPs. The size of the sliding window would affect the testing results. If the window size gets too large, excessive parameters need to be trained, which may add much extraneous information; if the window size is too small, it may not include enough essential information. The window size was selected to be five in this study. Empirically we found that such size was an appropriate choice for the model to have a solid performance.

#### Protein secondary structures

Protein secondary structure prediction (PSSpred version 2.0), a neural network classifier taken from the famous I-TASSER server, was utilized to predict the secondary structure of a peptide [45]. Each amino acid in an AMP was classified into α-helix, β-sheet, or random coil.

#### Conserved protein domains

Pfam domains based on multiple sequence alignments [46] were selected to represent known conserved protein domains. Each sequence was checked against Pfam. The regions mapped to the protein domains were labeled as conserved. Otherwise, not conserved.

#### Short Pairwise alignments

We designed an alignment procedure to search against the AMP core database. Neither global sequence alignment nor local sequence alignment was utilized, for this type of alignment focuses on long similar hits. Instead a gapless alignment using BLOSUM62 matrix was performed to search shorter similar hits, where only positive and neutral amino acid substitution were allowed. In addition, a heuristic approach by standardizing the alignment score by sequence length was used to rank the hits.

#### AMPA

AMPA was utilized to locate AMP stretches [33]. The default parameters were used to determine whether each amino acid resided in antimicrobial domains or not.

#### Aggregation

AGGRESCAN was utilized to estimate the aggregation tendencies of a peptide [47]. AGGRESCAN, which applied aggregation propensities of amino acids derived from the experimental data of β-amyloid peptides, was a good indicator of *in vivo* aggregation.

#### Net charges

A N-mer sliding window was utilized to go through the sequence. There were three categories of residues: leading, positive, and non-positive. All of the beginning N-1 residues were leading. The net charge of the N-mer is the difference between the count of the positive charged residues and that of the negative charged residues. Here N was set to 7.

#### Amphipathicity

Amphipathic moment, also known as the mean hydrophobic moment, was utilized to measure the amphipathicity of a peptide (S1 Equation) [48]. The amphipathicity of a peptide is that along the internal axis of the peptide backbone, one side contains non-polar residues and the other side contains polar or charged residues. Amphipathic moment quantifies such character by calculating the average hydrophobic difference of the two sides along the axis. HMOMENT with the default parameters from the EMBOSS version 6.5.7 was used to determine whether the peptide segment is amphipathic [49].

### Evaluation

The following statistical measures were utilized to evaluate model performance. They are sensitivity, specificity, accuracy, and Matthew’s coefficient of correlation (MCC) defined as follows:

$$\begin{array}{l} Sensitivity=\frac{TP}{TP+FN}\\ Specificity=\frac{TN}{FP+TN}\\ Accuracy=\frac{TP+TN}{TP+TN+FP+FN}\\ MCC=\frac{TP\times TN-FP\times FN}{\sqrt{\left(TP+FP)(TP+FN)(TN+FP)(TN+FN\right)}} \end{array}$$

TP, TN, FP, and FN are true positives, true negatives, false positives, and false negatives, respectively. Sensitivity is called the true positive rate; specificity is also known as the true negative rate. Accuracy is the percentage of the correction predictions among all the positive and negative data. MCC, which could range from −1 to 1, is a good performance indicator for a binary classifier. The larger the MCC value, the better the classifier.

## Results

The comparisons of the AMP cores and source proteins, which consist of the AMP critical and non-critical regions, were performed. The performance of our CRF models were then evaluated.

### Amino acid composition of the critical regions of AMPs

**Fig 3:**
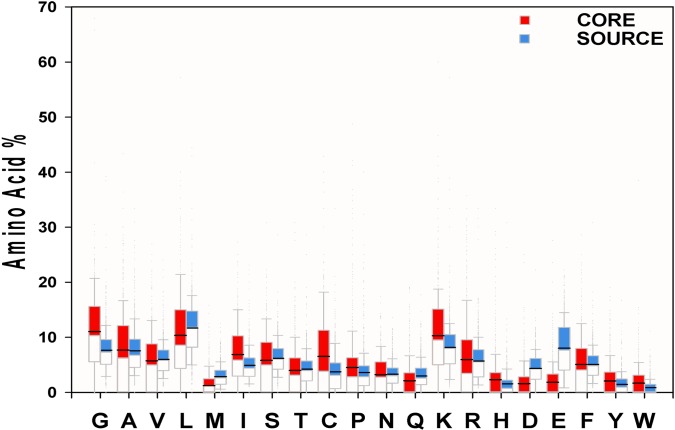
Statistical distribution of the amino acid composition of AMP critical regions and the background. The black line represents “mean”.

The comparison of amino acid composition of the critical regions of AMPs was shown in Fig. 3. Compared to the source proteins, the AMP cores showed higher percentages of glycine and lysine, but lower percentages of aspartic acid, glutamic acid, and methionine using the means and medians. The results by the means and medians were consistent except for arginine. For arginine, the AMP cores had a higher mean but much lower median than the source proteins. Interestingly, glycine, the smallest but flexible residue, was the most abundant residues in the critical regions of AMPs. In addition, the AMP cores and the source proteins shared similar hydrophobic contents. The difference of the total percentage of the hydrophobic residues between the AMP cores and the source proteins was negligible although about two-fifths of the overall residues were hydrophobic.

### Secondary structures of the critical regions of AMPs

**Fig 4:**
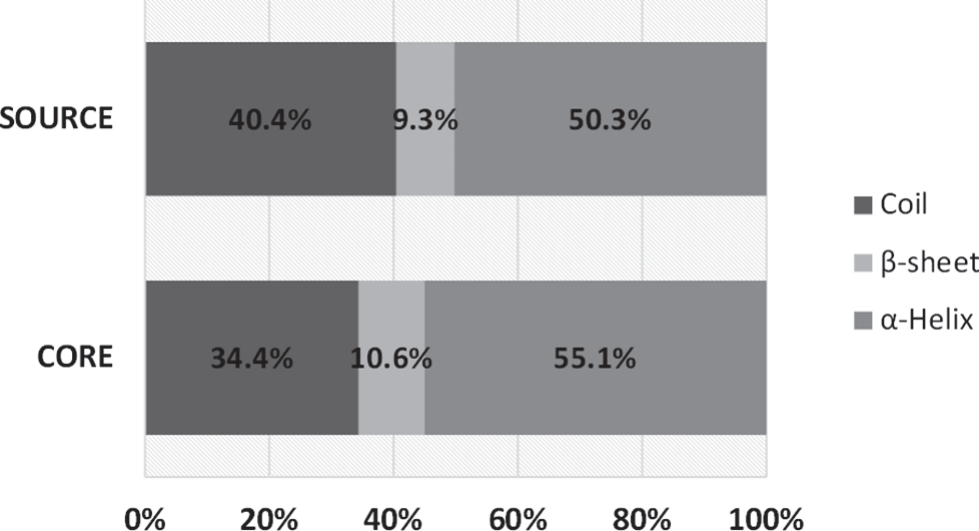
Statistical distribution of the secondary structures of AMP critical regions and the background.

The statistical analysis of protein secondary structures of the AMP cores was performed as shown in Fig. 4. The protein secondary structures were classified into α-helix, β-strand, or coil. More than half of AMP cores were α-helix, which was higher than the source proteins. In addition, the AMP cores had lower tendency to be coil than the source proteins. β-strand structures occurred infrequently in both the AMP cores and source proteins.

### Net charges of the critical regions of AMPs

**Fig 5:**
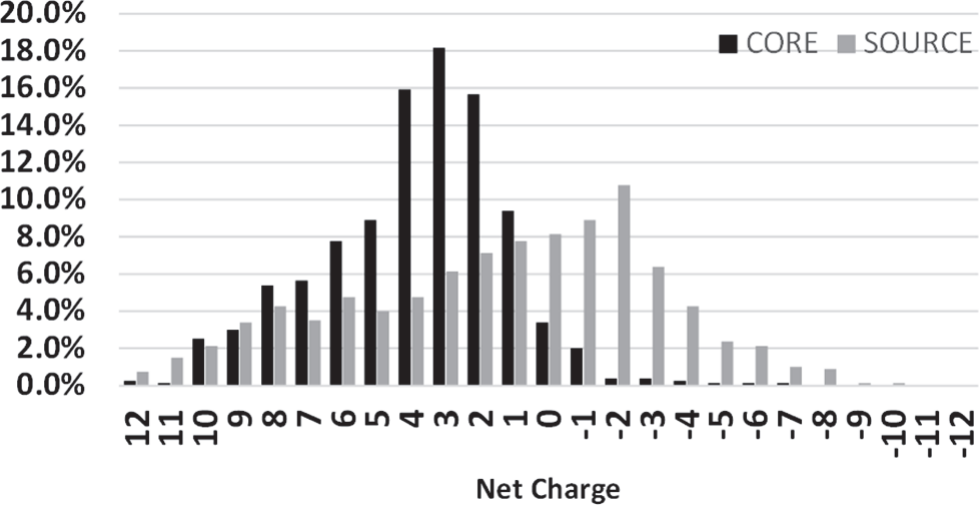
Statistical distribution of the net charge of AMP critical regions and the background. Less than 3% of the AMP critical regions and source proteins (background) having net charge over ±12 is not shown here.

The net-charge distributions of the AMP cores and the source proteins were compared in Fig. 5. The net-charge plot of AMP cores is similar to that of AMPs albeit with some slight differences [50]. The AMP cores predominantly had a positive net charge. About half of the AMP cores had a net charge between +2 and +4 and less than 5% of the AMP cores had a negative net charge. Compared to the AMP cores, the source proteins did not display a strong net-charge preference. Contrarily over one third of the source proteins had a negative net charge.

### Amphipathicity of the critical regions of AMPs

**Fig 6:**
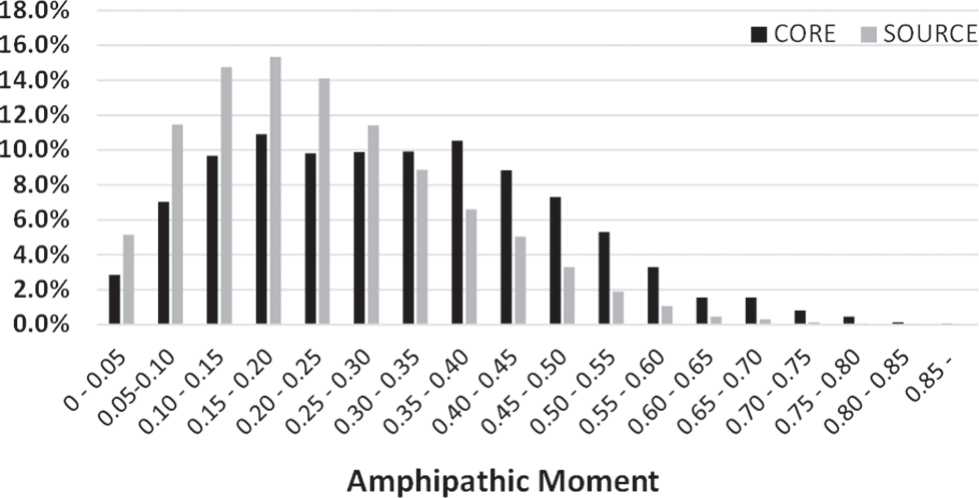
Statistical distribution of the amphipathicity of AMP critical regions and the background.

Fig. 6 compared the amphipathicity distributions of the AMP cores and the source proteins in terms of α helixes. The result showed that the AMP cores tended to have higher amphipathic values than the source proteins, suggesting that the AMP cores fit amphipathic α-helical structures better. However, the two amphipathicity distributions were less sufficiently separated than the two net-charge distributions in Fig. 5.

### Sequence motifs of the critical regions of AMPs

**Fig 7:**
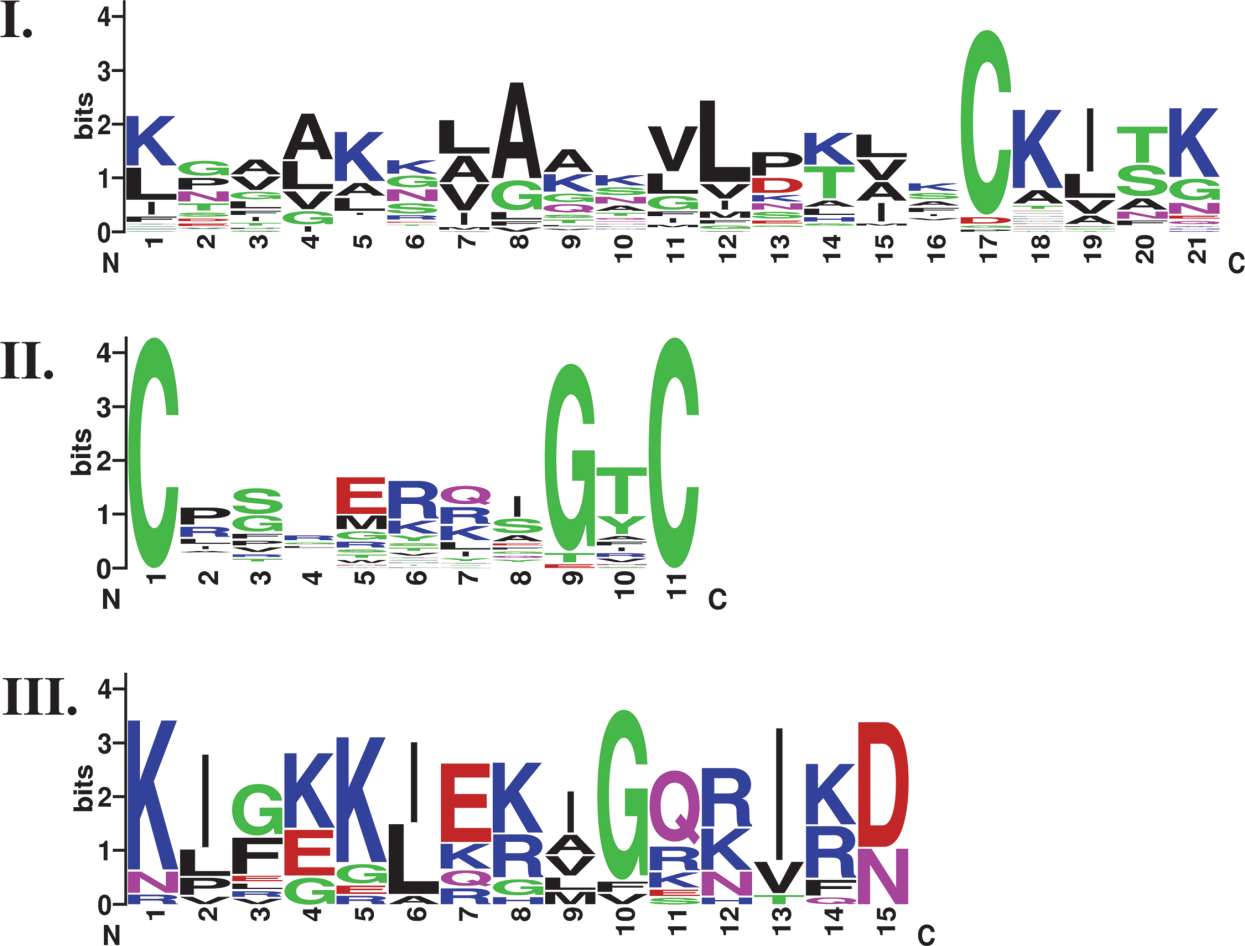
Sequences motifs of the AMP critical regions. Both motif I and III display periodic occurrences of positively charged residues. The helical wheel plots indicate that motif I fits into an amphipathic **π**-helix (S1 Fig.) and motif III is an amphipathic **α**-helix (S2 Fig.). Motif II is a cysteine-based motif.

The sequence motifs of the AMP cores were examined using MEME version 4.9.1 with the default parameters [51]. Three motifs were obtained as shown in Fig. 7. Two of the three motifs showed periodic occurrences of positively charged residues and the other was a cysteine-based motif. Both of the periodically charged motifs were further examined on a helical wheel plot. Interestingly they demonstrated a clear amphipathic property while one suited better into an amphipathic π-helical structure (S1 Fig.) and the other was a fine amphipathic α helix with one hydrophobic side and one hydrophilic side dominated with the positive charges (S2 Fig.). The cysteine-based motif acted like a hinge. The conserved cysteines at the edges of the cysteine-based motif were found to involve in the formation of disulfide bridges, not to each other, but to other distant cysteines; the glycine at the P9 could make the hinge motif more flexible.

### Boundaries of the critical regions of AMPs

**Fig 8:**
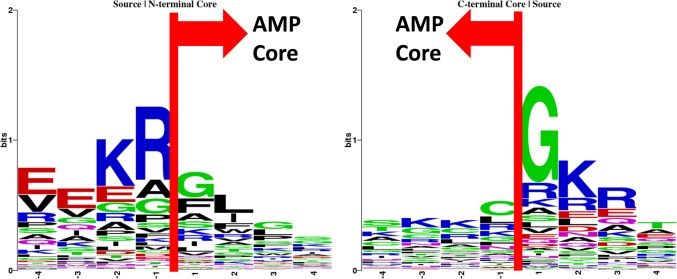
Sequences logos of the boundaries of the N-terminal and C-terminal AMP critical regions.

The N- and C-terminal boundaries of the AMP cores were examined as shown in Fig. 8. Just outside the N-terminal ends of the AMP cores (P-1), a weak preference for charged residues such as arginine was observed. Our analysis further showed the boundary arginine frequently paired with glycine, alanine, serine, and phenylalanine at the N-terminal end of the AMP cores. Particularly, arginine at the P-1 with glycine at the P1 occurred the most (S3 Fig.). In addition, adjacent to the C-terminal end of the AMP cores, glycine and positively charged residues were preferred. However, we found that the boundary bias at the C-terminal AMP cores was less apparent. More details could be found at S4 Fig.

### Performance comparison of CRF models

To evaluate the importance of each feature, the feature ablation studies were performed, which removes one of the testing features at a time. All the CRF models in the ablation studies were examined using 10-fold cross-validation on the testing datasets. Table 1 and Table 2 showed the performance comparison of the CRF models on the datasets of 798 AMP cores and 510 representative AMP cores respectively.

**Table 1:** Feature ablation study of AMPcore on the comprehensive dataset using 10-fold cross-validation.

Features	Sensitivity	Specificity	Accuracy	MCC
All	89.1	**90.1**	**89.6**	**0.79**
− Primary structures	73.5	88.0	80.8	0.62
− Secondary structures	88.4	89.1	88.8	0.78
− Net charges	88.9	88.9	88.9	0.78
− Amphipathicity	88.3	90.0	89.1	0.78
− Conserved domains	88.4	89.5	88.9	0.78
− Aggregation	88.9	89.3	89.1	0.78
− Gapless alignments	**89.3**	89.6	89.5	**0.79**
− AMPA	89.0	**90.1**	**89.6**	**0.79**

The symbol ‘−’ stands for “subtracting”.

**Table 2:** Features ablation study of AMPcore on the low similarity dataset using 10-fold cross-validation.

Features	Sensitivity	Specificity	Accuracy	MCC
All	82.3	84.0	**83.2**	**0.66**
− Primary structures	65.3	82.0	73.7	0.48
− Secondary structures	81.2	82.0	81.6	0.63
− Net charges	81.7	82.5	81.1	0.64
− Amphipathicity	81.9	82.2	82.0	0.64
− Conserved domains	81.9	82.5	82.2	0.65
− Aggregation	82.2	83.6	82.9	**0.66**
− Gapless alignments	**82.4**	84.0	**83.2**	**0.66**
− AMPA	81.3	**84.3**	82.8	**0.66**

The symbol ‘−’ stands for “subtracting”.

Not all of our testing features contribute to the model performance. Table 1 and Table 2 indicate that among these testing features, amino acid sequence, protein secondary structure, net charge, and amphipathicity were vital. However, aggregation, gapless alignment, and AMPA seemed less important, for they improved the CRF models little or none. Our CRF models could reach a maximal 90% accuracy and 0.79 MCC on the dataset of 798 AMP cores, but dropped to a maximal 83% accuracy and 0.66 MCC on the dataset of 510 representative AMP cores.

### Performance comparison of the AMP prediction models in predicting AMP cores

To verify if predicting AMP cores could be simply substituted by predicting AMPs, several AMP prediction models from CAMP webserver were investigated, including SVM, random forest, artificial neural network, and discriminant analysis [29]. Unlike our AMPcore models using these eight features built under 10-fold cross-validation, all the CAMP models were built on the full set of CAMP data with all of the AMP cores. A 10-mer sliding window, the minimum window size allowed in CAMP, was then utilized for these AMP models to go through the test cases of the AMP cores. As long as the predictors assigned the peptide segments to be AMPs, they would be marked as the critical regions. On the comprehensive dataset, the best CAMP models could achieve a maximal accuracy of 70% and 0.40 MCC; on the low similarity dataset, they could reach a maximal accuracy of 68% and 0.37 MCC (Table 3 and Table 4).

**Table 3:** Performance comparison with AMP prediction models on the comprehensive dataset of 798 AMP cores.

Model	Sensitivity	Specificity	Accuracy	MCC
**CAMP.SVM**	55.0	59.8	56.7	0.14
**CAMP.RF**	47.0	47.9	47.5	-0.05
**CAMP.ANN**	62.7	82.2	68.4	0.41
**CAMP.DA**	71.6	68.2	69.7	0.40
**AMPcore**	89.1	90.1	89.6	0.79

**Table 4:** Performance comparison with AMP prediction models on the low similarity dataset of 510 AMP cores.

Model	Sensitivity	Specificity	Accuracy	MCC
**CAMP.SVM**	54.9	60.1	56.8	0.15
**CAMP.RF**	47.4	49.0	48.2	-0.04
**CAMP.ANN**	60.5	78.9	65.7	0.35
**CAMP.DA**	70.1	67.0	68.4	0.37
**AMPcore**	82.3	84.0	83.2	0.66

## Discussion

This was the first study to systematically extract, examine, and model the critical regions of AMPs. Several properties of the AMP cores were found to be common with those of the AMPs, but the boundary bias of the AMP cores were newly discovered in this study. We demonstrated that the general-purposed AMP prediction tools were not suited for the prediction of AMP cores and our *ad hoc* CRF model with multiple features would work better.

Several key features are common to both AMPs and AMP cores. The AMP cores are abundant in glycine and lysine, but deficient in methionine and acidic residues, the same as the AMPs [1]. The N-terminal ends of the AMP cores are relatively rich in glycine, similar to the findings in antibacterial peptides [28]. Besides, as cationic AMPs prevail, the AMP cores are predominantly having a positive net charge. Further, the periodic occurrences of positively charged residues lead to the amphipathicity of the AMP cores, also known to the AMPs [10]. Like what have been suggested in AMPs, these properties might help the AMP cores to interact with anionic microbial cell membranes.

There are also novel findings reporting for the first time. Plenty of charged residues were found right outside the N-terminal and C-terminal ends of the AMP cores. These charged residues might help stabilize the AMPs attached to microbes. In addition, we discovered that R/G, R/F, R/S, and R/A at the boundary of the N-terminal AMP cores occurred more frequently than would be expected by chance. Particularly, R/G was the most apparent.

Why arginine at the P-1 of the source proteins coupling with glycine at the P1 of the N-terminal AMP cores is not clear. While AMP cores remain antimicrobial activities, the boundaries of AMP cores might provide accessory functions. Two possible explanations are proposed: (1) The R/G site might be a cell adhesion signal to microbes, similar to the RGD peptides of fibronectin, an adhesion protein, to cell hosts [52]. Fibronectin-binding protein might be one of the receptors for the naturally occurring AMPs. (2) The R/G site might involve with the interaction of RNA, for RG-rich protein domains are known to affect RNA binding [53]. Additional studies, which are beyond the scope of this study, are still required to determine the role of R/G at the boundary of the N-terminal AMP cores.

Other than the boundary preferences, the secondary structures of the AMP cores were quantified. Most of the AMP cores were α-helix, a common structure for protein-protein interactions, which was similar to our previous finding in antiviral peptides [54]. Besides, the AMP cores possessed a stronger amphipathic helical character than the background. Two amphipathic sequence motifs within the AMP cores were found: One α-helix and one π-helix. A previous study even suggested that relatively infrequent π-helix was directly linked to the active sites of proteins [55]. Interestingly, our findings of the AMP cores supported this assertion.

Predicting AMP cores could not be replaced simply by predicting AMPs. Table 3 and Table 4 demonstrate that by far our CRF models of AMP cores outperformed these current AMP prediction models. CRFs were chosen to build AMPcore, for they are generally superior in modeling the sequential data [36]. In fact, in order to reach optimal performances in predicting AMP cores, our CRF model required not only amino acid sequences but also additional features such as protein secondary structures, net charges, and amphipathicity. Accurately predicting AMP cores in protein sequences would facilitate experimental designs.

## Supporting Information

S1 Fig Amphipathic π-helical wheel plot of an AMP core containing motif I. (TIF)

S2 Fig Amphipathic α-helical wheel plot of an AMP core containing motif III. (TIF)

S3 Fig Relative distribution of amino acid coupling at the N-terminal boundary of AMP cores. Each row represents the residue at the P(-1) of the boundary of the source protein; Each column represents the residue at the P1 of the N-terminal AMP cores. Each cell represents the log-odd value for such pattern against the background. A heat map is used to scale the log-odd value: low (black) to high (white).
(TIF)

S4 Fig Relative distribution of amino acid coupling at the C-terminal boundary of AMP cores. Each row represents the residue at the P(-1) of the C-terminal AMP cores; Each column represents the residue at the P1 of the boundary of the source proteins. Each cell represents the log-odd value for such pattern against the background. A heat map is used to scale the log-odd value: low (black) to high (white).
(TIF)

S1 Equation Amphipathic moment. (DOCX)
