# Supplementary figures and images for: Analysis and Prediction of the Critical Regions of Antimicrobial Peptides Based on Conditional Random Fields
Source: PLoS One. 2015 Mar 24;10(3):e0119490. doi: 10.1371/journal.pone.0119490 (PMC4372350; doi:10.1371/journal.pone.0119490)

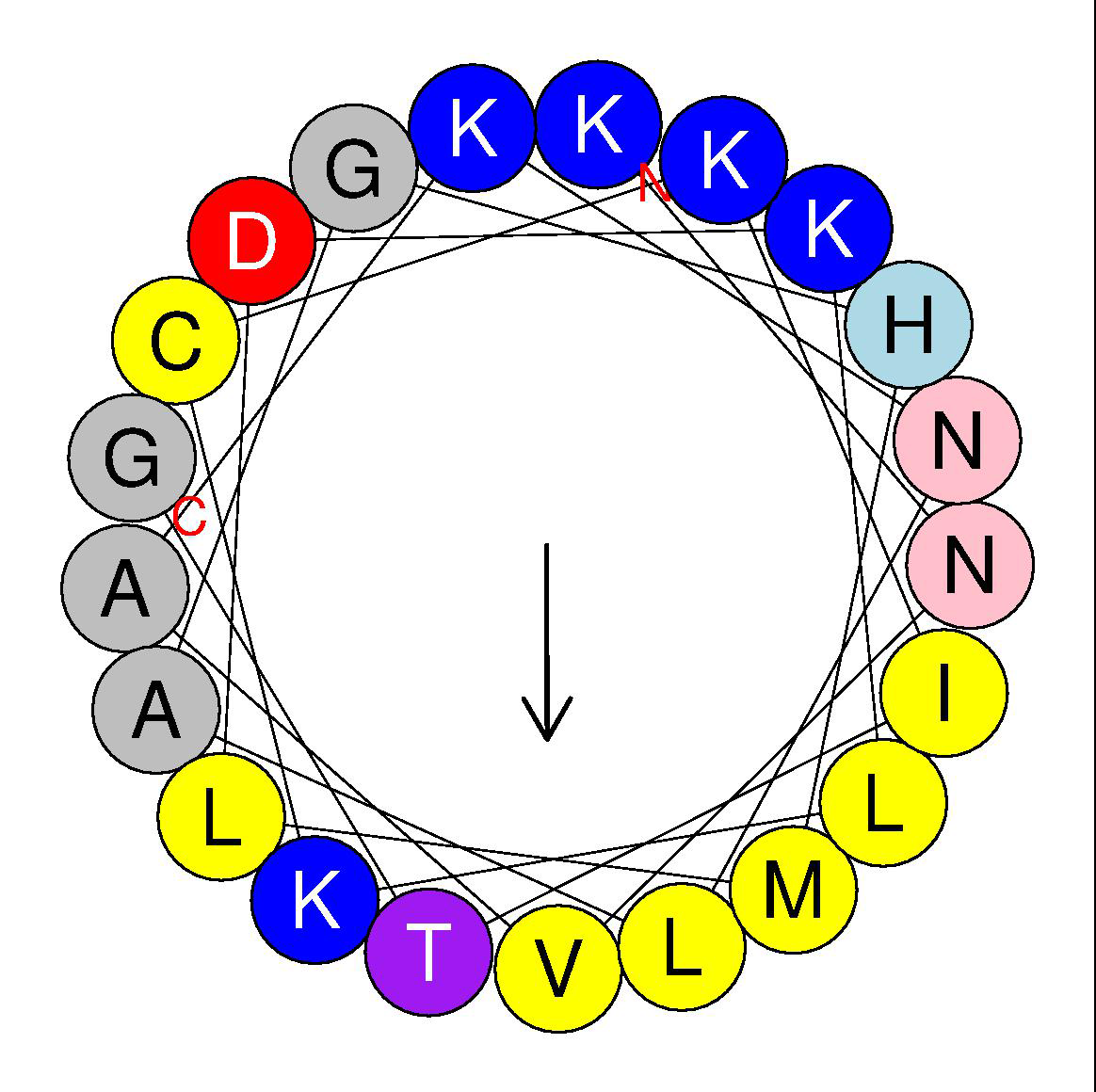

Supplement: S1 Fig — (TIF) [file pone.0119490.s001.tif]

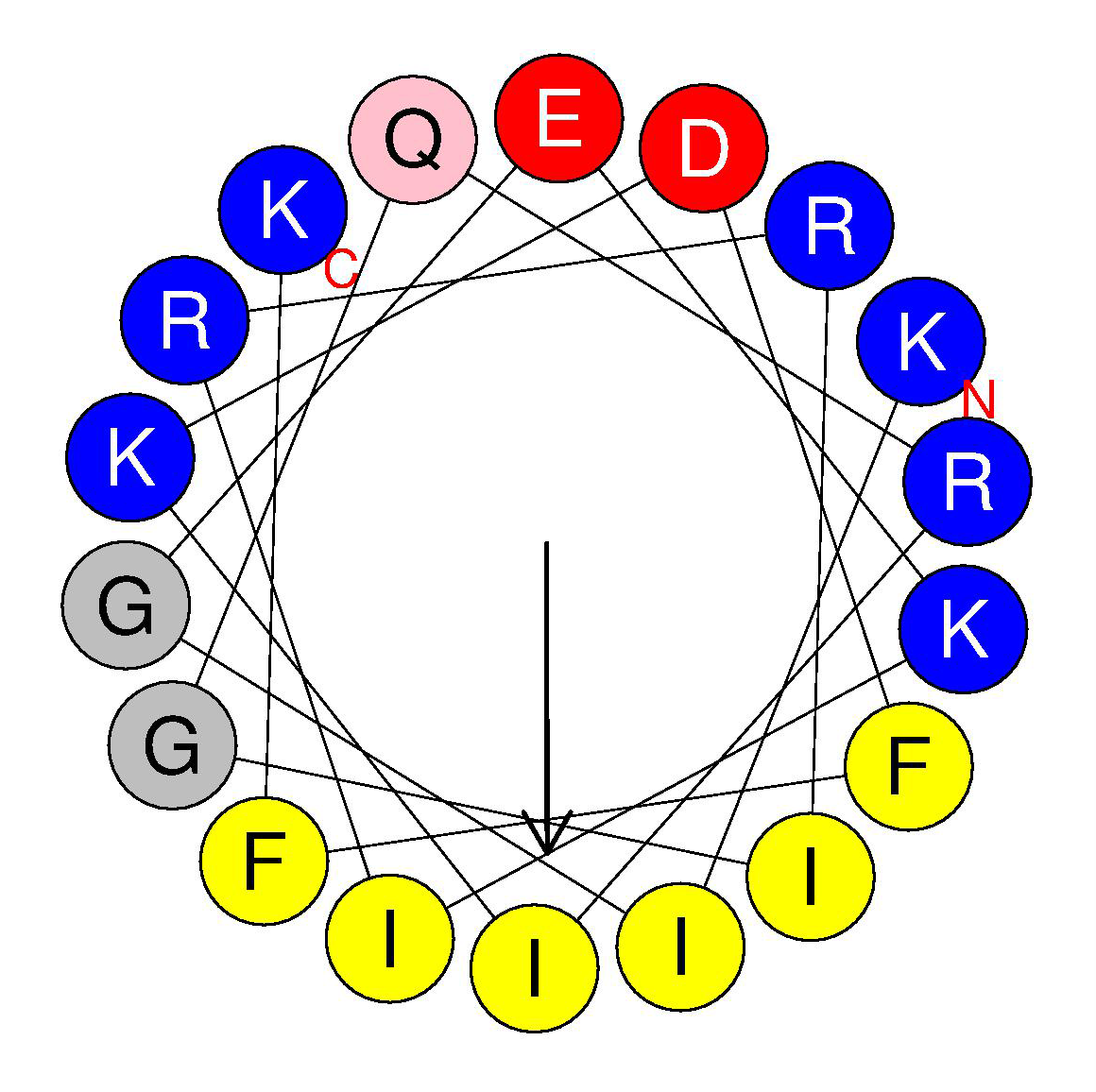

Supplement: S2 Fig — (TIF) [file pone.0119490.s002.tif]
