# Supplementary material for: Analysis and Prediction of the Critical Regions of Antimicrobial Peptides Based on Conditional Random Fields
Source: PLoS One. 2015 Mar 24;10(3):e0119490. doi: 10.1371/journal.pone.0119490 (PMC4372350; doi:10.1371/journal.pone.0119490)
Supplement: S1 Equation — (DOCX) [file pone.0119490.s005.docx]

**Equation S1.** Amphipathicity moment

$$A_{m}=\bar{\mu H}=\frac{1}{N}\sqrt{{[\sum_{n=1}^{N} H_{n}\sin(n\delta)]}^{2}+{[\sum_{n=1}^{N} H_{n}\cos(n\delta)]}^{2}}$$

where *N* is the peptide length, *H_n_* is the hydrophobic value of the *n*th residue of the peptide, and δ is the angle along the internal axis of the peptide backbone. The larger the values, the greater amphipathicity the peptide is.
